# The outcome of arthroscopic repair of acetabular labral tears using the iHOT-33

**DOI:** 10.1186/s12891-019-2611-3

**Published:** 2019-05-13

**Authors:** Jesse Renouf, Nicholas Pergaminelis, Phong Tran, Camdon Fary, Oren Tirosh

**Affiliations:** 1 Department of Orthopaedic Surgery, Western Health, Melbourne, Victoria 3011 Australia; 2 Australian Institute for Musculoskeletal Science (AIMSS), St Albans, Melbourne, VIC Australia; 3 The University of Melbourne and Western Health, Parkville, Melbourne, VIC Australia

**Keywords:** Hip arthroscopy, Patient reported outcomes, Acetabular labrum

## Abstract

**Background:**

The purpose of this study was to determine patient reported outcome measures (PROMS) after arthroscopic repair of an isolated labral tear using the validated International Hip Outcome Tool (iHOT-33). The iHOT-33 specifically measures (1) symptoms and functional limitations, (2) sport and recreation limitations, (3) job related concerns and social and (4) emotional and lifestyle concerns.

**Methods:**

A retrospective review was performed on 45 procedures in 43 patients between September 2012 and September 2015. Two patients had bilateral isolated labral tears. Patients were excluded if they were younger than 18 years, had prior ipsilateral hip surgery and had radiological or arthroscopic evidence of femoroacetabular impingement (FAI), hip dysplasia or other bony dysmorphism.

**Results:**

Of the 43 patients undergoing arthroscopy there were 29 right and 16 left hips repaired. There were 34 females and 9 males. The mean age at surgery was 37.4 years (range 19–63 years) with a mean follow up of 1.7 years (range 1.0–2.6 years). At follow up the mean total iHOT-33 score improved from 34.1 to 67.3 (*p* < 0.02). The mean improvement was 33.2 (p = < 0.02). Significant improvements were described in all 4 iHOT-33 sub sections.

**Conclusion:**

The study showed statistically significant favourable outcomes in selected patients with short follow-up for patients that underwent hip arthroscopy for an isolated labral tear using the validated iHOT-33.

**Level of Evidence:**

IV, retrospective non-randomised study.

## Background

The acetabular labrum has a role in shock absorption, joint lubrication, pressure distribution and improves stability by deepening the acetabulum [1, 2]. A torn labrum compromises these functions, with acetabular labral lesions being associated with early degenerative joint disease [3]. Arthroscopic management of a labral tear has been shown to relieve pain and improve function with a low morbidity and complication rate [4, 5].

Outcome studies have utilized a variety of patient reported outcome measures (PROMS) including; the Hip Outcome Score (HOS), Non-Arthritic Hip Score (NAHS), Harris Hip Score (HHS) and Western Ontario and McMaster Universities Osteoarthritis index (WOMAC). Many of which have ceiling effects and comparatively poor psychometric properties in regards to newer validated scores such as the International Hip Outcome Tool (i-HOT-33) [6, 7]. Critical appraisal of development, measurement properties and comparison studies suggest the iHOT-33 is currently the most validated tool for hip preservation surgery in young and active patients [6].

The purpose of the study was to investigate the outcome of primary repairs of labral tears in patients without an underlying osseous abnormality using the validated iHOT-33 outcome score with minimum 1-year post-operative follow-up.

## Methods

A retrospective review of our hip arthroscopy database was performed. There were 2541 patients included in the database from hip arthroscopies performed by two surgeons between September 2012 and September 2015. Of these patients 459 had labral repairs. Patients were excluded if they were younger than 18 years, had less than one-year follow-up, had prior ipsilateral hip surgery and had radiological or arthroscopic evidence of femoroacetabular impingement (FAI), hip dysplasia or other bony dysmorphism.

After exclusion criteria was applied there were 71 procedures in 69 patients who received primary repair of a torn acetabular labrum. Of these there were 26 patients failed to respond postoperatively via email or post. Thus the final sample size was 43 patients undergoing 45 procedures.

Indication for surgery in all patients was recalcitrant hip pain and associated mechanical symptoms that were not responsive to conservative treatment for at least 6 months. On clinical examination, all patients had positive pain provocation tests (flexion, adduction and internal rotation) and were investigated using plain radiographs (AP pelvis, lateral, Dunn view), magnetic resonance imaging scans (MRI) and CT scans to exclude osseous abnormality including femoroacetabular impingement and hip dysplasia.

### Surgical technique

Hip arthroscopy was performed under general anaesthesia in the lateral decubitus position using a McCarthy Hip Distractor (Innomed, Inc., Savannah, GA, USA). With the use of an image intensifier two portals were established; a viewing portal above the apex of the greater trochanter and an instrumentation portal 3-4 cm anterior to the anterior margin of the greater trochanter. An arthroscopic pump was used throughout to maintain constant distension of the joint with Ringer solution, at a pressure of 40 mmHg. A 70° arthroscope was used throughout the procedure in both the central and peripheral compartments.

Classification of an acetabular labral tear was described intraoperativley based on location of the lesion on the labral circumference using a clock-face nomenclature where 6 o’clock was the transverse ligament and 3 o’clock was anterior [8, 9]. The Labrum was then treated with circumferential suture anchor refixation using the Stryker Nano Tack Flex (Kalamazoo, MI, USA).

Upon visualization of the central compartment, articular cartilage pathology was classified according to the Outerbridge classification and The international cartilage research society (ICRS) grading system, [10, 11]. Microfracture was undertaken in patients with full-thickness cartilage loss (Outerbridge grade 4) at the chondrolabral junction in lesions up to 3 cm2.

During the arthroscopy procedure a dynamic inspection of the LT using both internal and external rotation was performed. Ligamentum teres (LT) tears were described using the Salas and O’Donnell classification system [12]. LT tears were debrided with a radiofrequency probe (Vulcan Eflex Ablator Probe, Smith & Nephew, And over, MA, USA). Local anaesthetic (100 mg ropivicaine) was injected via portals into the hip joint and the wounds were closed with interrupted Nylon sutures 2.0.

Surgical findings were recorded at the time of surgery. All patients underwent formal rehabilitation post-operatively using a standard protocol [13]. Post-operatively all patients were discharged with crutches, full weight bear status and instructed to take oral meloxicam for 30 days.

### Outcomes score

Each patient was asked to complete the iHOT-33 questionnaire prior to surgery on consultation. Post operatively patients completed the i-HOT33 via email or post. The iHOT-33 is a self-administered tool containing 33 questions distributed within four domains; symptoms and functional limitations, sports and recreational activities, job-related concerns and social, emotional and lifestyle concerns. Each question is answered on a visual analogue scale format ranging from 0 to 100, where a higher score represents a higher quality of life.^7^ The total score is the mean score for each item. Questions included may not be applicable to all patients and have the option to not to be answered. These optional questions relate to cutting/changing direction during sports activities, job related concerns, sexual activities and carrying children. If these questions are omitted or unable to be answered then the overall score is still taken as the average out of 100 from all the questions answered.

### Statistical analysis

Multivariate model with repeated measures was used to explore differences between the iHOT-33 pre-surgery and post-surgery total scores. LT-tear (yes or no) and chondroplasty (yes or no) were used as the between-subject effects to determine if they had effect on the outcome. We correlated patients with ligamentum teres tear or chondroplasty using Bonferroni tests. The iHOT-33 total score was the within-subjects effects (repeated effects). Repeated measures were also performed on the 4 iHOT-33 domains; symptoms, sport, job, and social. All statistical tests were run using SPSS version 24 (IBM® SPSS Statistics) with α set at 0.05, *p* < 0.05, as the level of significance.

## Results

Of the 43 patients undergoing arthroscopy there were 29 right and 16 left hips repaired. The majority of the patients were female (34 females, 9 males). The mean age at surgery was 37.4 years (95% CI 19–63 years) with a mean follow up of 1.7 years (95% CI 1.0–2.6 years). Only 26.7% of patients could recall an obvious event or trauma causing their pain. Patients were involved in common sports included dancing, running, triathlons and weightlifting (Table 1).

**Table 1:** Patient Demographics

Patient demographics	
Number of patients	43
Mean age	37.41
Number of hips	45
Male: female	10:35
Left: Right	16:29
Etiology	
Non-specific	31
Motor-vehicle accident	3
Lifting injury	3
Running injury	3
Wakeboarding injury	1
Dancing Trauma	1
Fall	1
Sports/Activity	
Dancing	6
Running	5
Triathlons	4
Weightlifting	4
Lifting injury	2
Australian Football	2
Motor vehicle accident	2
Rugby	1
Netball	1
Swimming	1
Wake-boarding	1

The majority of labral tears were anterior in 40 hips (88.9%) and superior in 5 hips (11.1%) (Table 2). Labral tears were identified intraoperatively using the clock face method of localization and were between the 10 o’clock and 2 o’clock position. The mean labral tear size was 1.78 clock face spacing’s (1–5).

**Table 2:** Operative Findings

	Number	Percent
Labral tear location		
anterior	40	88.9
superior	5	11.1
Ligamentum Teres tear	35	77.8
Grade 0	10	22.2
Grade 1	6	13.3
Grade 2	0	0.0
Grade 3	15	33.3
Grade 4	1	2.2
Grade 5	13	28.9
Grade 6	0	0.0
Ligamentum teres ablation	35	77.8
Chondropathy		
*OuterBridge*		
Grade 0	8	20.0
Grade 1	19	42.2
Grade 2	17	37.8
Grade 3	1	2.2
Grade 4	0	0.0
*ICRS*		
Grade 0	8	17.8
Grade 1	22	48.9
Grade 2	14	31.1
Grade 3	1	2.2
Grade 4	0	0.0
Chondroplasty	28	62.2

Associated chondral damage was present in 37 hips (82.2%). They were classified according to the Outerbridge classification and The international cartilage research society (ICRS) grading system [10, 11]. 35 hips (77.8%) had partial tears of the LT, which underwent radiofrequency ablation. LT tears were classified using the arthroscopic classification proposed by Sales & O’Donnell. [12] No correlation between change in i-HOT33 score and ligaemtnum teres was shown (*p* = 0.52). Similarly, i-HOT33 score change was not affected in those patients who required chondroplasty(*p* = 0.15).

At follow up the mean total iHOT-33 score improved from 34.1 (95% CI 16.8–51.4) to 67.3 (95% CI 44.7–89.9), *p* < 0.02). The mean improvement was 33.2 (p = < 0.02). In addition, significant improvements were described in all 4 iHOT-33 sub sections (Table 3). The minimum clinically important difference for the i-HOT33 is 6.1. [7]

**Table 3:** Pre and Post operative i-HOT 33 scores

	Mean Pre-Operative Score	Mean Post-Operative Score	Mean Change	p
Section 1: Symptoms & Functional Limitations	40.1	72.1	32.0	0.019
Section 2: Sports & Recreational Activities	19.0	50.6	31.6	0.020
Section 3: Job-Related Concerns	41.7	69.6	27.9	0.020
Section 4: Social, Emotional & Lifestyle Concerns	27.6	61.5	33.9	0.020
TOTAL SCORE	34.1	67.3	33.2	0.019

## Discussion

The majority of labral tears are associated with other bony pathology such as FAI. Isolated labral tears occur less frequently and often result from a significant traumatic event or repetitive trauma or iliopsoas impingement [14, 15]. This is generally secondary to the labrum’s weight bearing role at extremes of joint range [16]. Regardless of the cause impaired labral function can result in significant pain, reduced function and can impair a patient’s quality of life.

In our cohort, patients showed significant short-term improvement in all functional domains of the iHOT-33 with mean follow up of 1.7 years. These studies are consistent with other results in the literature that also show modest improvements [6, 17–22]. Although only few examine isolated tears directly [20, 21]. In 2011 Haviv and O’Donnell noted improvements when evaluating labral repairs without bony dysmorphism using the MHHS and NAHS. In their study of 81 patients the MHHS improved by 18 points and the NAHS by 17 points [20].

There are multiple PROMS used in the literature to assess patient outcome following hip arthroscopy. Older PROMS show ceiling effects and comparatively poor psychometric properties in respect to newer and more validated scores such as the iHOT-33. Despite this the Modified Harris Hip Score (MHHS) is still the most common PROM used to assess hip arthroscopic repair of acetabular labral tear [23]. Other PROMS such as the HHS and WOMAC are also used but were originally designed for hip arthroplasty and hip osteoarthritis rather than hip arthroscopy [24, 25]. Multiple studies compare current PROMS and the iHOT-33 has shown to have superior reliability, construct validity and responsiveness for use in patients undergoing hip preservation surgery in young and active patients [26]. This is the first study that uses iHOT-33 to assess acetabular labral tear repair.

Arthroscopy allows visualisation of nearby articular structures, in particular, the articular cartilage and ligamentum teres. Acetabular labral tears are associated with articular cartilage lesions and usually occur at the watershed zone at the labrochondral junction [27]. In this study 82.2% of the patients had associated acetabular chondral rim lesions and of these 73.7% requiring chondroplasty. These results are similar to other studies that show an association with articular pathology. Mcarthy et al. found that 63% of hip arthroscopy for labral tears showed chondral damage [28]. Kamath et al. required 40% of patients to undergo concomitant chondroplasty.^19^ Recently Stake et al. suggested patients with articular cartilage damage have worse outcomes [18]. Other studies have reported no effect on outcomes are at least shown conflicting results. [29, 30]. Our study did not show any significant correlation with chondral damage and worse patient outcomes at 1 year. However, longitudinal follow-up is required to clarify the prognostic value of cartilage damage after arthroscopic repair of labral tears.

In our study 35 hips (77.8%) had partial tears of the ligamentum teres, which all underwent radiofrequency ablation. The ligamentum teres tightens in external rotation, and may have a secondary stabilizer role with labral deficiency [31]. If the ligament is torn it can cause impingement and be a source of disabling pain and are usually debrided to a stable remnant [32]. In this study, the presence and degree of tearing of ligamentum teres tears had no effect on outcome.

Patients with pre-existing osteoarthritis have poorer outcomes after hip arthroscopy for labral pathology [1, 22, 26, 33, 34]. Meftah et al. revealed that only 19% of patients with arthritic changes had a good or excellent outcome. Compared to 62% for the total patient cohort [22]. This study showed no statistical correlation on chondral damage and patient outcome. This could be due to the low prevalence of significant cartilage damage in our cohort. A systematic review used to grade indications of hip arthroscopy showed poor-quality conflicting evidence regarding the use of hip arthroscopy for the treatment of mild to moderate osteoarthritis of the hip [35]. It is likely that given the patient cohort size and age that a larger and older cohort would improve the assessment on labral tear repair in the setting of chondral damage.

Although the distinctive strength of this study is using a validated PROM (i-HOT33) on specific patients without bony dysmorphism; there are still limitations. The inherent limitations of retrospective studies include selection bias, loss to follow up, lack of control subjects and limited sample size. This study had significant non respondents which could also overestimate the positive effect. In addition, the study has a relatively short term follow up, as the iHOT33 is a recently available outcome score. However, we considered that 1 year minimum follow up time is a reasonable time to assess the early results of symptomatic relief and complications. Longer-term follow-up will be useful and we are conducting a longer follow up on these patients.

## Conclusion

The study showed statistically significant favourable short-term outcomes in patients undergoing hip arthroscopy for a hip labral tear without bony dysmorphism using the validated iHOT-33.

## Abbreviations

ALAD: The international cartilage research society
FAI: Femoroacetabular impingement
HHS: Harris Hip Score
HOS: Hip Outcome Score
ICRS: The international cartilage research society
i-HOT33: International Hip Outcome Tool
LT: Ligamentum Teres
MAHORN: Multicenter Arthroscopic Hip Outcomes Research Network
MHHS: Modified Harris Hip Score
NAHS: Non-Arthritic Hip Score
PROMS: Patient reported outcome measures
WOMAC: Western Ontario and McMaster Universities Osteoarthritis index

## References

[1] Ferguson SJ, Bryant JT, Ganz R, Ito K (2000). The influence of the acetabular labrum on hip joint cartilage consolidation: a poroelastic finite element model. J Biomech.

[2] Ferguson SJ, Bryant JT, Ganz R, Ito K (2000). The acetabular labrum seal: a poroelastic finite element model. Clin Biomech.

[3] Altenberg AR (1977). Acetabular labrum tears: a cause of hip pain and degenerative arthritis. South Med J.

[4] Lynch TS, Terry MA, Bedi A, Kelly BT (2013). Hip arthroscopic surgery: patient evaluation, current indications, and outcomes. Am J Sports Med.

[5] Robertson WJ, Kadrmas WR, Kelly BT (2007). Arthroscopic Management of Labral Tears in the hip: a systematic review. Clin Orthop Relat Res.

[6] Ramisetty N, Kwon Y, Mohtadi N (2015). Patient-reported outcome measures for hip preservation surgery—a systematic review of the literature. Journal of hip preservation surgery.

[7] Mohtadi NG, Griffin DR, Pedersen ME, Chan D, Safran MR, Parsons N, Sekiya JK, Kelly BT, Werle JR, Leunig M, McCarthy JC (2012). The development and validation of a self-administered quality-of-life outcome measure for young, active patients with symptomatic hip disease: the international hip outcome tool (iHOT-33). Arthroscopy: The Journal of Arthroscopic & Related Surgery.

[8] McCarthy JC, Noble P, Schuck M, Aluisio FV, Wright J, Lee JA (2003). Acetabular and labral pathology. InEarly hip disorders.

[9] Schmerl M, Pollard H, Hoskins W (2005). Labral injuries of the hip: a review of diagnosis and management. J Manip Physiol Ther.

[10] Outerbridge RE. The etiology of chondromalacia patellae. J Bone Joint Surg Br. 1961;43. 10.1302/0301-620X.43B4.752 14038135

[11] Brittberg M, Winalski CS (2003). Evaluation of cartilage injuries and repair. JBJS.

[12] Porthos Salas A, O’Donnell JM (2015). Ligamentum teres injuries-an observational study of a proposed new arthroscopic classification. Journal of hip preservation surgery.

[13] Takla A (2009). The hip: assessment, arthroscopy and the TOP protocol. Sports Physio Australia.

[14] Tanzer M, Noiseux N (2004). Osseous abnormalities and early osteoarthritis: the role of hip impingement. Clin Orthop Relat Res.

[15] Domb BG, Shindle MK, McArthur B, Voos JE, Magennis EM, Kelly BT (2011). Iliopsoas impingement: a newly identified cause of labral pathology in the hip. HSS J.

[16] Groh MM, Herrera J (2009). A comprehensive review of hip labral tears. Current reviews in musculoskeletal medicine.

[17] Jackson TJ, Hammarstedt JE, Vemula SP, Domb BG (2015). Acetabular labral base repair versus circumferential suture repair: a matched-paired comparison of clinical outcomes. Arthroscopy: The Journal of Arthroscopic & Related Surgery.

[18] Stake CE, Jackson TJ, Stone JC, Domb BG (2013). Hip arthroscopy for labral tears in workers’ compensation: a matched-pair controlled study. Am J Sports Med.

[19] Kamath AF, Componovo R, Baldwin K, Israelite CL, Nelson CL (2009). Hip arthroscopy for labral tears. Am J Sports Med.

[20] Haviv B, O’Donnell J (2011). Arthroscopic treatment for acetabular labral tears of the hip without bony dysmorphism. Am J Sports Med.

[21] Streich NA, Gotterbarm T, Barié A, Schmitt H (2009). Prognostic value of chondral defects on the outcome after arthroscopic treatment of acetabular labral tears. Knee Surg Sports Traumatol Arthrosc.

[22] Meftah M, Rodriguez JA, Panagopoulos G, Alexiades MM (2011). Long-term results of arthroscopic labral debridement: predictors of outcomes. Orthopedics.

[23] Kemp JL, Collins NJ, Roos EM, Crossley KM (2013). Psychometric properties of patient-reported outcome measures for hip arthroscopic surgery. Am J Sports Med.

[24] Banaszkiewicz PA. Traumatic arthritis of the hip after dislocation and acetabular fractures: treatment by mold arthroplasty: an end-result study using a new method of result evaluation. InClassic Papers in Orthopaedics. 2014:13–7. 5783851

[25] Bellamy N, Buchanan WW, Goldsmith CH, Campbell J, Stitt LW (1988). Validation study of WOMAC: a health status instrument for measuring clinically important patient relevant outcomes to antirheumatic drug therapy in patients with osteoarthritis of the hip or knee. J Rheumatol.

[26] Byrd JT, Jones KS (2014). Primary repair of the acetabular labrum: outcomes with 2 years' follow-up. Arthroscopy: The Journal of Arthroscopic & Related Surgery.

[27] Singh PJ, O'Donnell JM (2010). The outcome of hip arthroscopy in Australian football league players: a review of 27 hips. Arthroscopy: The Journal of Arthroscopic & Related Surgery.

[28] McCarthy J, Noble P, Aluisio FV, Schuck M, Wright J, Lee JA (2003). Anatomy, pathologic features, and treatment of acetabular labral tears. Clin Orthop Relat Res.

[29] Farjo LA, Glick JM, Sampson TG (1999). Hip arthroscopy for acetabular labral tears. Arthroscopy: The Journal of Arthroscopic & Related Surgery.

[30] Byrd JT, Jones KS (2000). Prospective analysis of hip arthroscopy with 2-year follow-up. Arthroscopy: The Journal of Arthroscopic & Related Surgery.

[31] Rao J, Zhou YX, Villar RN (2001). Injury to the ligamentum teres. Clin Sports Med.

[32] Bharam S (2006). Labral tears, extra-articular injuries, and hip arthroscopy in the athlete. Clin Sports Med.

[33] Beck M, Kalhor M, Leunig M, Ganz R (2005). Hip morphology influences the pattern of damage to the acetabular cartilage. Bone & Joint Journal.

[34] Philippon MJ (2001). The role of arthroscopic thermal capsulorrhaphy in the hip. Clin Sports Med.

[35] Stevens MS, LeGay DA, Glazebrook MA, Amirault D (2010). The evidence for hip arthroscopy: grading the current indications. Arthroscopy: The Journal of Arthroscopic & Related Surgery.

